# Cell Cycle-independent Role of Cyclin D3 in Host Restriction of Influenza Virus Infection[Fn FN1]

**DOI:** 10.1074/jbc.M117.776112

**Published:** 2017-01-27

**Authors:** Ying Fan, Chris Ka-Pun Mok, Michael Chi Wai Chan, Yang Zhang, Béatrice Nal, François Kien, Roberto Bruzzone, Sumana Sanyal

**Affiliations:** From the ‡ HKU-Pasteur Research Pole and; § Centre of Influenza Research, School of Public Health, LKS Faculty of Medicine, University of Hong Kong, Hong Kong SAR, China,; the ¶ Medical Research Council Protein Phosphorylation and Ubiquitylation Unit, University of Dundee, Dundee DD1 4HN, Scotland, United Kingdom,; the ‖ Harbin Institute of Technology, Shenzhen Graduate School, Shenzhen, Guangdong 518055, China,; the ** Division of Biosciences, College of Health and Life Sciences, Brunel University London, London UB8 3PH, United Kingdom,; ‡‡ Ksilink, French-German Advanced Translational Center, Strasbourg 67000, France, and; the §§ Department of Cell Biology and Infection, Institut Pasteur, Paris Cedex 75015, France

**Keywords:** cell cycle, G1/S cyclin, influenza, protein degradation, protein-protein interaction, M2, cell cycle arrest, cyclin D3, restriction factor

## Abstract

To identify new host factors that modulate the replication of influenza A virus, we performed a yeast two-hybrid screen using the cytoplasmic tail of matrix protein 2 from the highly pathogenic H5N1 strain. The screen revealed a high-score interaction with cyclin D3, a key regulator of cell cycle early G_1_ phase. M2-cyclin D3 interaction was validated through GST pull-down and recapitulated in influenza A/WSN/33-infected cells. Knockdown of *Ccnd3* by small interfering RNA significantly enhanced virus progeny titers in cell culture supernatants. Interestingly, the increase in virus production was due to cyclin D3 deficiency *per se* and not merely a consequence of cell cycle deregulation. A combined knockdown of *Ccnd3* and *Rb1*, which rescued cell cycle progression into S phase, failed to normalize virus production. Infection by influenza A virus triggered redistribution of cyclin D3 from the nucleus to the cytoplasm, followed by its proteasomal degradation. When overexpressed in HEK 293T cells, cyclin D3 impaired binding of M2 with M1, which is essential for proper assembly of progeny virions, lending further support to its role as a putative restriction factor. Our study describes the identification and characterization of cyclin D3 as a novel interactor of influenza A virus M2 protein. We hypothesize that competitive inhibition of M1-M2 interaction by cyclin D3 impairs infectious virion formation and results in attenuated virus production. In addition, we provide mechanistic insights into the dynamic interplay of influenza virus with the host cell cycle machinery during infection.

## Introduction

The threat of influenza infection is felt globally, and the disease leads to an estimated 3–5 million cases of severe illness and about 250,000–500,000 deaths each year (1). Although the strategy of antagonizing influenza infection through vaccination has been partially successful, the necessity for effective antiviral agents is underscored by the toxic side effects of currently available drugs and the emergence of drug-resistant variants (2–4). Influenza viruses, like any other viruses, are obligate intracellular pathogens that exploit the host cellular machinery to replicate. The identification of cellular mechanisms required for the virus life cycle not only sheds light on its trafficking characteristics but also provides strategies for interfering with host factors as an alternative approach to inhibit viral infection.

Elucidating host-pathogen interactions as an unbiased approach to gain insights into disease pathogenesis has received ample attention. Various functional assay systems have been used for screening host factors involved in the influenza virus replication cycle thus far. From RNA interference (RNAi)-based genome-wide screening strategies in *Drosophila* (5) and mammalian systems (6–9) to other approaches, including proteomics (10, 11), yeast two-hybrid (Y2H)^3^ analyses (7, 12), and microarrays (7, 13–15), extensive information has been acquired. Multiple cellular networks have been implicated in influenza virus replication through either direct protein-protein interactions (10, 16) or signaling pathways (17). Although these strategies unveiled hundreds of genes important for the virus life cycle, their functional relevance and molecular mechanisms are still poorly understood. Among others approaches, effort has been invested to dissect how the cellular interactors of viral ribonucleoprotein complexes regulate the replication and transcription of influenza virus (18). Host interactors of the non-structural protein 1 (NS1), a multifunctional protein modulating several aspects of the virus replication cycle with a major role in inhibiting interferon mediated immune response, have also been extensively studied (16). However, little attention has been drawn to identifying cellular factors associated with the viral matrix protein 2 (M2).

We reasoned that the integral membrane proteins of the viral envelope would interact with cellular factors at various stages: endosomal fusion and release of the genetic material during entry, transport from endoplasmic reticulum to the plasma membrane, and assembly and budding of nascent virions. M2 is a minor protein of the viral envelope that forms a homotetramer in its native state (19, 20). Interestingly, M2 possesses the longest C-terminal tail among the three viral envelope proteins, namely hemagglutinin (HA), neuraminidase, and M2. It is an ion channel that was initially discovered as the target of the antiviral drug amantadine and facilitates diffusion of protons to the interior of the endosomally entrapped virus (21). Low pH induces a conformational change in HA and subsequently triggers fusion with the endosomal membrane during virus entry (22). M2 is a 97-residue single-pass membrane protein that displays considerable pleiotropism. It determines the filamentous morphology of some viral strains through binding to cholesterol (23–25). The cytoplasmic tail (CT) of M2 interacts with M1 at the site of virus budding for efficient packaging of virus particles (26, 27). Rossman *et al.* (28) reported a role of M2-CT in mediating cholesterol-dependent alteration in membrane curvature at the neck of budding virions, leading to host ESCRT pathway-independent membrane scission.

Altogether, these studies provide evidence that influenza M2, especially the CT domain, plays a critical role in multiple steps of the virus life cycle. Hence, the identification of cellular interactors of M2 would provide mechanistic insights into influenza pathogenesis and possibilities for development of novel strategies to interfere with multiple steps of the infection process. By using M2-CT as bait, we screened a human placenta complementary DNA (cDNA) library to identify host proteins that either facilitate or restrict viral infection. Cyclin D3, a key regulator of cell cycle G_0_/G_1_ phase progression, was uncovered as a novel host factor interacting with M2-CT. The physical interaction between M2 and cyclin D3 was confirmed in virus-infected cells. Influenza A virus (IAV) infection resulted in host cell cycle arrest in G_0_/G_1_ phase, which was accompanied by cyclin D3 relocalization and degradation. Using a combination of small interfering RNA (siRNA)-mediated genetic analyses we further showed that cyclin D3 restricts IAV production, independent of its role in the cell cycle. The restriction of cyclin D3 on IAV life cycle did not impair viral protein synthesis but interfered with M1-M2 binding, which may result in defective assembly and release of progeny virions. The role of cyclin D3 in the context of influenza infection has not been described previously. More interestingly, our results suggest a novel function of cyclin D3 that is beyond its classical function in cell cycle regulation.

## Results

### 

#### 

##### Identification of Cyclin D3 as M2-CT-binding Protein

**FIGURE 1.**
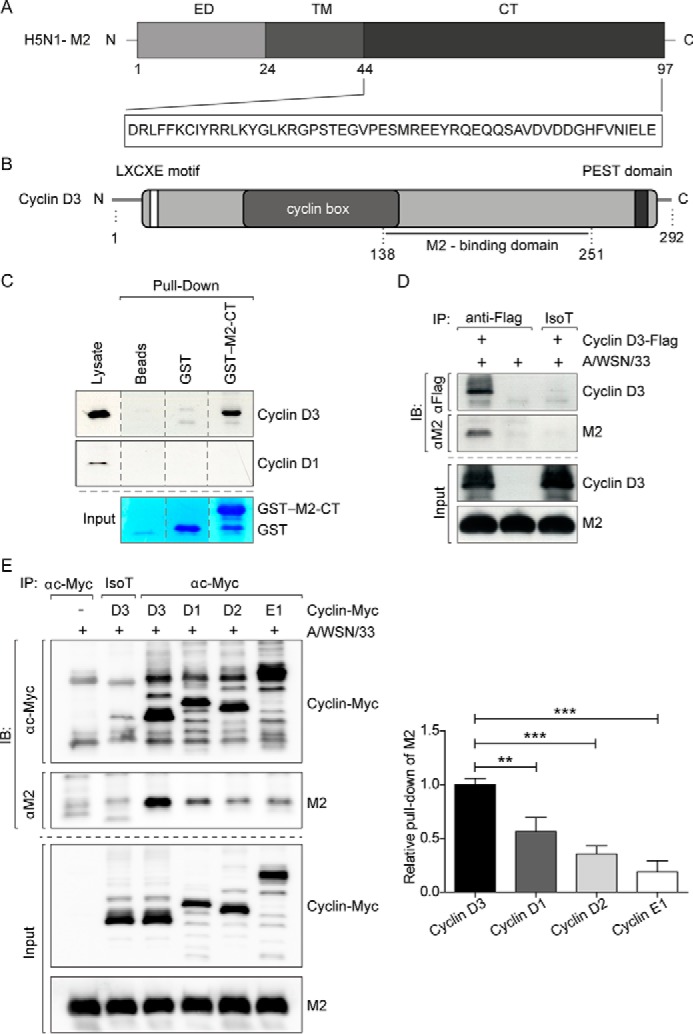
**Identification of cyclin D3 as a specific cellular interactor of M2 influenza protein.** *A*, schematic representation of structural motifs in the M2 protein with the enlarged sequence of the CT used as the bait for the yeast two-hybrid screen. *ED*, ectodomain; *TM*, transmembrane domain. *B*, schematic representation of cyclin D3. The retinoblastoma (Rb)-binding L*X*C*X*E motif; the proline (P), glutamic acid (E), serine (S), and threonine (T)-rich PEST domain, which has been postulated to be involved in the rapid degradation of unstable proteins; and the M2-minimal interacting domain are shown. *Numbers*, amino acid positions. *C*, GST pull-down assay using whole cell lysates from A549 cells. Samples were incubated with either naked beads, GST control (*GST*), or GST fused to the cytoplasmic tail of A/WSN/33 virus M2 protein (*GST-M2-CT*) and analyzed by immunoblotting with anti-cyclin D3 antibodies after pull-down. Results are representative of three and two independent experiments for cyclin D3 and cyclin D1 pull-down, respectively. *Dotted lines*, sliced lanes from the same gel. GST protein input was revealed by Coomassie Blue staining. *D*, co-immunoprecipitation of M2 with cyclin D3. HEK 293T cells infected with A/WSN/33 virus at an MOI of 2 were lysed at 6 h after infection and subjected to immunoprecipitation (*IP*) with anti-FLAG or isotype control antibodies. Cell lysates or pellets following immunoprecipitation were analyzed by immunoblotting (*IB*) using the indicated antibodies. Transfection and infection conditions are shown *above* the *blots*. Results are representative of three independent experiments. *E*, HEK 293T cells were transfected with plasmids encoding Myc-tagged cyclins 48 h before infection with A/WSN/33 virus at an MOI of 2. Cells were lysed at 6 h after infection and subjected to immunoprecipitation with anti-Myc or isotype control antibodies. Cell lysates or pellets following immunoprecipitation were analyzed by immunoblotting using the indicated antibodies. Transfection and infection conditions are shown *above* the *blots*. M2 was found to interact with all cyclins tested, albeit to different intensities. Results are representative of three independent experiments. Quantitation of M2 pull-down levels was performed by densitometric analysis of immunoblots. M2 level was normalized to the respective immunoprecipitated Myc-tagged protein level in each condition. The degree of association of M2 with cyclin D1 was around 50% that of cyclin D3 when the same amount of cyclins was immunoprecipitated. Weak binding of M2 to cyclin D2 and cyclin E1, which plays a role in the cell cycle clock one step later than D-type cyclins and is required for cell cycle G_1_/S transition in association with cyclin-dependent kinase 2 (CDK2), was also observed in our experiments. The bar graph shows the mean ± S.D. (*error bars*) of three independent experiments. **, *p* < 0.005; ***, *p* < 0.0001 by one-way ANOVA.

The IAV M2 ion channel protein is a multifunctional protein with a highly conserved sequence among influenza A virus isolates that approaches 95% identity in some regions (29, 30). Among the three viral envelope proteins, M2 possesses the longest CT with a high probability of interactions with the cellular machinery at various steps of the virus life cycle. These include fusion, intracellular trafficking through the secretory pathway (31), and virus assembly and budding at the plasma membrane (24, 32). It is of considerable interest to identify intracellular interactors of M2-CT, not only to elucidate cellular components that are exploited by influenza, but also to understand host defenses involved in restricting the virus life cycle. To this end, a random-primed cDNA library from human placenta was screened by Y2H using the IAV M2-CT as bait (Fig. 1*A*). The screening revealed human cyclin D3 (GenBank^TM^ accession number NM_001760.2) as one of the most prominent interactors of M2-CT, as summarized in Table 1. Sequence alignment of the positive clones pointed to amino acid residues 138–251 as the minimal interacting domain for M2-CT (Fig. 1*B*). The M2-binding domain on cyclin D3 is largely outside of its cyclin box, which is responsible for binding to and activation of cyclin-dependent kinases (33, 34) and also the region in which the greatest homology occurs among the D-type cyclins (35). This finding, together with the absence of other cyclins among positive clones, suggests a certain degree of specificity of the interaction, which was tested in the next series of experiments.

**TABLE 1:** **Yeast two-hybrid screen identifies/predicts cyclin D3-M2-CT interaction**

Characteristic	Value or description
Bait	M2 cytoplasmic tail (amino acids 44–97)
Library	Random-primed human placenta cDNA
No. of interactions tested	63.89 million
Prey	Cyclin D3
No. of positive clones	42
Confidence score*^a^*	A
Minimal interacting domain	Amino acids 138–251

*^a^* Predicted biological score (from A to E, highest to lowest), a statistical confidence score assigned to each interaction by Hybrigenics.

Cyclin D forms a complex with and functions as a regulatory subunit of cyclin-dependent kinases 4 and 6 (CDK2 and -4), whose activity is required in the cell cycle early G_1_ phase (36). There are three early G_1_ phase D-type cyclins (D1, D2, and D3), which are functionally related with tissue-specific expression profiles (37–43). Our results from Y2H screening indicate that only cyclin D3, and not the other two D-type cyclins, interacts with M2-CT. We first checked the specificity of this interaction in human A549 lung epithelial cells. GST-M2-CT fusion protein was purified and incubated with wild-type cell lysate to pull down endogenous D-type cyclins. Cyclin D3 bound specifically to GST-M2-CT and not to either GST or beads (Fig. 1*C*). Altogether, these results confirm that cyclin D3 binds to the IAV M2-CT.

To examine the intracellular association of M2-CT with cyclin D3 during the IAV life cycle, co-immunoprecipitation experiments were performed from virus-infected cells. HEK 293T cells were transiently transfected with a plasmid encoding FLAG-tagged cyclin D3 (cyclin D3-FLAG) and infected with influenza A/WSN/33 virus. Cell lysates were immunoprecipitated with anti-FLAG antibody and immunoblotted with anti-M2 antibody. As shown in Fig. 1*D* (*lane 1*), M2 was found to co-purify with cyclin D3, demonstrating their physical interaction during virus infection, either directly or through a common interactor. No interaction was observed in control samples, where cells were either mock-transfected and immunoprecipitated with anti-FLAG antibody (Fig. 1*D*, *lane 2*) or transfected with cyclin D3-FLAG and immunoprecipitated with isotype control antibody (Fig. 1*D*, *lane 3*). These data validate cyclin D3 as a *bona fide* interactor of influenza M2, corroborating our results from the Y2H screening.

The specificity of interaction between cyclin D3 and M2 was tested using a panel of exogenously expressed cyclins (cyclin D1, D2, D3, and E1). Although M2 appears to interact with other cyclins to some degree when they are overexpressed, we consistently found that it has the strongest interaction with cyclin D3 (Fig. 1*E*).

##### IAV Infection Induces Cyclin D3 Relocalization and Targets It for Proteasomal Degradation

**FIGURE 2.**
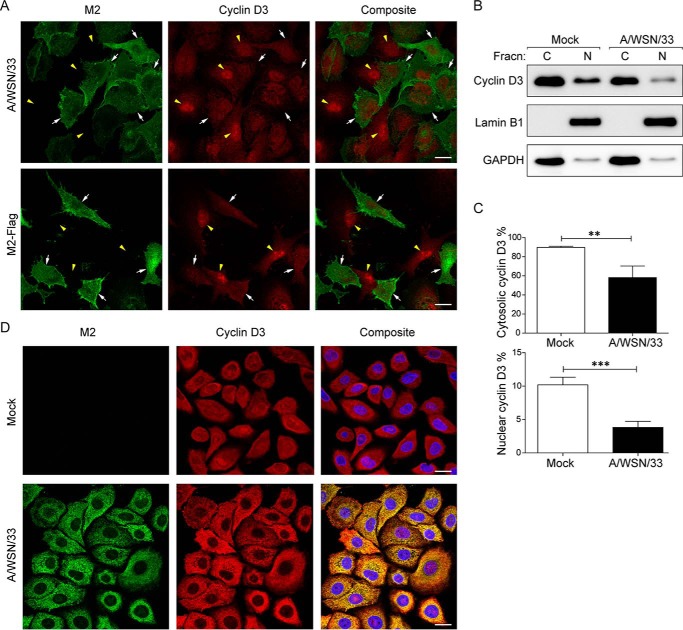
**Influenza A virus infection drives cyclin D3 redistribution from nucleus to cytoplasm.** *A*, cyclin D3 subcellular distribution is altered upon M2 expression. A549 cells were infected with A/WSN/33 virus at an MOI of 0.5 or transfected with a plasmid expressing M2 protein. At 24 h after infection or transfection, cells were fixed for immunofluorescence staining. The subcellular localization of cyclin D3 (*red*) and M2 (*green*) was visualized through immunostaining with specific antibodies and analyzed by confocal microscopy. *Yellow arrowheads*, nuclei of uninfected or non-transfected cells. *White arrows*, virus-infected or M2-expressing cells. *Bar*, 20 μm. *B*, cyclin D3 translocates from nucleus to cytoplasm after viral infection. *Left*, cytoplasmic (*C*) and nuclear (*N*) fractions of virus-infected (MOI of 2) or uninfected cells were prepared from A549 cells 8 h after infection. Lysates were resolved by SDS-PAGE and immunoblotted with anti-cyclin D3. Levels of lamin B1 and GAPDH were used as controls to verify clean separation of nuclear and cytoplasmic fractions, respectively. Results are representative of three independent experiments. *C*, quantitation of cyclin D3 levels was performed by densitometric analysis of immunoblots. Data are presented as percentage of cyclin D3 remaining in the nucleus as a fraction of the total. Both mock- and virus-infected groups were normalized to total cyclin D3 levels in uninfected cells. The bar graph shows the mean ± S.D. (*error bars*) of three independent experiments. **, *p* < 0.005 by unpaired Student's *t* test. *D*, colocalization of cyclin D3 and M2 in virus-infected cells. A549 cells expressing cyclin D3 were infected with A/WSN/33 virus at an MOI of 2 in the presence of MG-132 to block protein degradation through proteasome and fixed at 15 h postinfection. The subcellular localization of cyclin D3 (*red*) and M2 (*green*) was visualized through immunostaining with specific antibodies and analyzed by confocal microscopy. *Bar*, 20 μm.

To understand the functional relevance of cyclin D3-M2 interaction in virus-infected cells, we tested their subcellular distribution through immunofluorescence imaging. To achieve unbiased analysis, A549 cells were infected with influenza A/WSN/33 virus at an MOI of 0.5 to have a mixed population of infected and uninfected cells in the same sample. Cells were fixed at 24 h postinfection (p.i.) and stained for cyclin D3 and M2 proteins. In uninfected cells, cyclin D3 resides both in the nucleus and in the cytoplasm with only the nuclear CDK4/6-cyclin D3 complex displaying retinoblastoma (Rb)-directed kinase activity (44). Interestingly, upon virus infection, a much weaker cyclin D3 signal was observed in the nuclei of infected cells compared with control cells, as revealed by M2 staining. These results suggest that cyclin D3 is redistributed from the nucleus to the cytosol as a response to infection (Fig. 2*A*, *top panels*). The effect of M2 protein on cyclin D3 relocalization was recapitulated in analogous experiments using A549 cells expressing M2 protein alone (Fig. 2*A*, *bottom panels*), which resulted in the apparent reduction of cyclin D3 from the nucleus, similar to the phenotype induced by viral infection.

To address the reduction of cyclin D3 from the nucleus upon virus infection, we fractionated both control and infected cells into cytosol and nuclear material to measure cyclin D3 levels. Cytoplasm- and nucleus-enriched extracts were prepared from A/WSN/33-infected (MOI of 2 to achieve a more homogeneous infection of the cell culture) and uninfected A549 cells. Lamin B1 and GAPDH were used as markers to verify clear separation of nuclear and cytoplasmic fractions. Total intracellular level of cyclin D3 in virus-infected cells decreased to ∼60% of that observed in mock-infected cells (Fig. 2, *B* and *C*, *top*). Densitometric analysis indicates that 10% of total cyclin D3 was found in the nucleus of uninfected cells. This is very likely an underestimation because cyclin D3, being soluble, would leak from the nuclear into the cytoplasmic fraction during the experimental procedures. The percentage of nuclear cyclin D3 dropped to <4% at 8 h p.i. (Fig. 2*C*, *bottom*), indicating translocation of cyclin D3 from the nucleus to the cytoplasm in virus-infected cells. This significant (>60%) reduction of nuclear cyclin D3 detected through biochemical measurement confirmed our immunofluorescence data on its subcellular relocalization.

We further verified colocalization of cyclin D3 with that of M2 through infection of A/WSN/33 in cyclin D3-expressing cells. Because at 24 h p.i. most of cyclin D3 is degraded, we selected an earlier time point and treated mock- and virus-infected cells with MG-132 to increase the signal strength of cyclin D3. Indeed, at 15 h p.i., we could capture colocalization of influenza M2 and cyclin D3 along the secretory pathway (Fig. 2*D*). Most of cyclin D3 appeared in the cytosolic fraction under these conditions. Interaction of cyclin D3 with M2, combined with its enrichment in the cytosol upon M2 expression strongly suggests that this association must occur in the cytosol, either directly or as a complex presumably en route to assembly and release of intact progeny virions.

**FIGURE 3.**
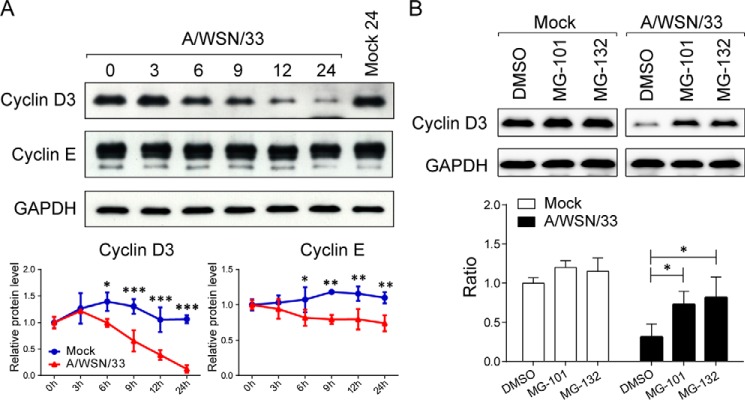
**IAV infection down-regulates protein levels of cyclin D3 through proteasome-mediated degradation.** *A*, IAV infection reduces protein level of cyclin D3 but not cyclin E. A549 cells were infected with A/WSN/33 virus at an MOI of 2. *Top*, cells were lysed at specified time points, subjected to SDS-PAGE, and visualized by Western blotting. Results are representative of three independent experiments. *Bottom*, densitometric quantitation of cyclin D3 and cyclin E normalized to GAPDH. Line graphs represent mean ± S.D. (*error bars*) from three independent experiments. *, *p* < 0.05; **, *p* < 0.01; ***, *p* < 0.001 *versus* mock at each time point by two-way ANOVA. *B*, cyclin D3 is targeted for proteolysis after viral infection. *Top*, A549 cells, either mock- or virus-infected, were treated with proteasome inhibitors MG-101 or MG-132 at the initiation of viral infection. Whole cell lysates were extracted at 9 h after infection for detection of cyclin D3 protein levels. *Bottom*, densitometric analysis of immunoblots for cyclin D3 levels (normalized to GAPDH). Results are representative of three independent experiments. The bar graph shows the mean ± S.D. from three independent experiments. *, *p* < 0.05 by unpaired Student's *t* test.

The induction of D-type cyclins is growth factor-dependent, and both mRNAs and proteins exhibit moderate oscillations in continuously proliferating cell populations (37, 38, 45–48). Given the decrease in total intracellular cyclin D3 levels observed in virus-infected cells, we next addressed whether influenza virus modulates cyclin D3 protein levels in addition to altering its subcellular localization. The expression profiles of cyclin D3 and another cell cycle G_1_ phase regulator, cyclin E, were measured through immunoblotting. Cyclin D3 was dramatically down-regulated by IAV infection to almost undetectable levels compared with uninfected cells at 24 h p.i. (Fig. 3*A*, *top*). A time course analysis revealed that the steady-state levels of cyclin D3 started declining at 6 h p.i. and decreased by ∼50% at 9 h p.i. (Fig. 3*A*, *bottom*). Cyclin E, which is activated at the late stage of cell cycle G_1_ phase to facilitate G_1_/S transition, displayed only a moderate reduction under identical conditions (Fig. 3*A*), contrary to the finding by He *et al.* (49), where cyclin E was vastly down-regulated by IAV infection. However, no quantitative analysis was provided in their study, preventing us from comparing the reduction level. It is worth noting that cyclin E exists as multiple proteins ranging in size from *M*_r_ 35,000 to 50,000 in tumor cell lines, as illustrated by others (see also Fig. 3*A*) (50, 51). However, only one band size of cyclin E was shown in their study, although the same cell type was used. The differential regulation of cyclin D3 and cyclin E indicates that IAV infection specifically deregulates components in the early G_1_ phase (G_0_/G_1_ phase) of the cell cycle machinery.

**FIGURE 4.**
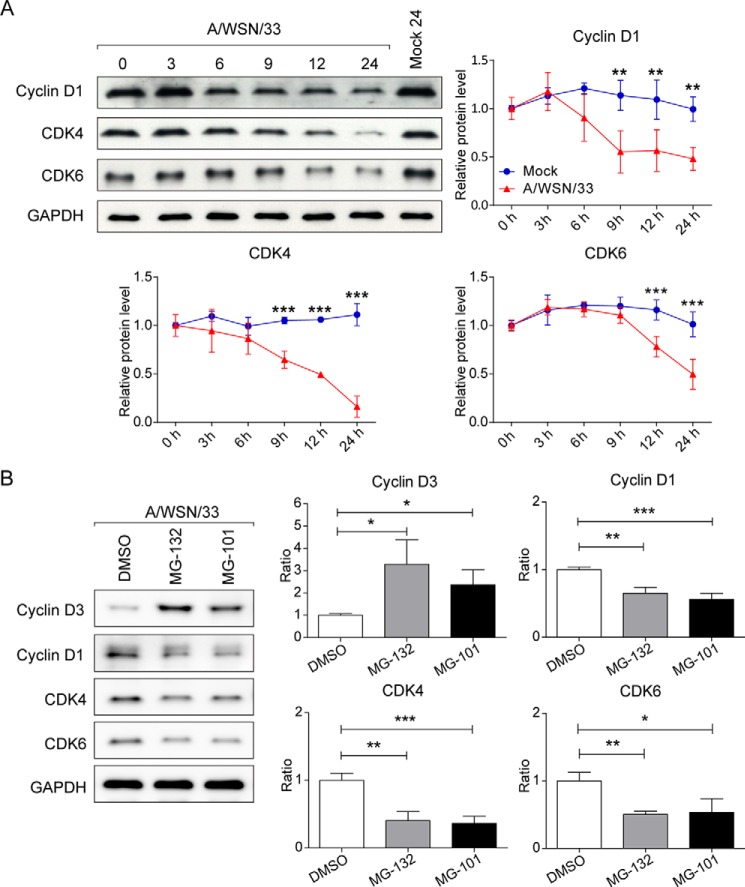
**IAV specifically targets cyclin D3 for proteasomal degradation.** *A*, IAV infection reduces protein level of cyclin D1, CDK4, and CDK6. A549 cells were infected with A/WSN/33 virus at an MOI of 2. Cells were lysed at specified time points, subjected to SDS-PAGE, and visualized by Western blotting. Results are representative of three independent experiments. Line graphs represent mean ± S.D. (*error bars*) of band intensity obtained through densitometric quantitation from three independent experiments. **, *p* < 0.01; ***, *p* < 0.001 *versus* mock at each time point by two-way ANOVA. *B*, cyclin D3 is specifically targeted for proteolysis after viral infection. Virus-infected (MOI of 2) A549 cells were treated with proteasome inhibitors MG-101 or MG-132 at the initiation of viral infection. Whole cell lysates were extracted at 24 h p.i. for detection of the protein levels of cell cycle regulators. Results are representative of three independent experiments. The bar graph shows the mean ± S.D. of band intensity obtained through densitometric analysis from three independent experiments (normalized to GAPDH). *, *p* < 0.05; **, *p* < 0.005; ***, *p* < 0.001 by unpaired Student's *t* test.

To further investigate the mechanism of virus-induced cyclin D3 down-regulation, we determined the involvement of the proteasomal degradation pathway in this process. Cells were infected with A/WSN/33 virus in the absence or presence of proteasome inhibitors, MG-101 or MG-132, and tested for cyclin D3 levels. MG-101, also known as calpain inhibitor I, inhibits both proteasome and calpain activities (52). Calpain is a calcium-dependent protease that produces limited proteolysis of its substrates. Resulting fragments are further degraded by other proteolytic pathways, including proteasome (52). Calpain has been reported to regulate cyclin D1 at the posttranscriptional level (53). MG-132 is a proteasome inhibitor that prevents degradation of ubiquitin-conjugated proteins (54). Uninfected cells were treated with the same drugs to control for the baseline oscillation of cyclin D3 levels. Proteasome inhibition did not affect cyclin D3 abundance in uninfected cells; however, blocking proteasome activity led to a significant stabilization of cyclin D3 in virus-infected cells. MG-101 and MG-132 treatments blocked cyclin D3 degradation by ∼50 and ∼60%, respectively, compared with the vehicle control (DMSO)-treated group, as measured by densitometric analysis of immunoblots (Fig. 3*B*). Thus, an obvious rescue of cyclin D3 was observed after one round of infection (9 h p.i.) through the use of proteasome inhibitors. The regulation of other cell cycle moderators was also investigated. Although viral infection also resulted in cyclin D1, CDK4, and CDK6 down-regulation on protein level (Fig. 4*A*), the level of down-regulation is not as robust as that of cyclin D3, and their protein levels could not be rescued by protease and calpain inhibitor when compared with cyclin D3 (Fig. 4*B*). These findings suggest that IAV posts a cyclin D3-specific effect during infection.

Our data indicate that IAV infection results in reduced cyclin D3 levels in the nucleus of virus-infected cells. This phenotype is triggered by translocation of nuclear cyclin D3 into the cytoplasm, where IAV facilitates proteasomal degradation of cytoplasmic cyclin D3.

##### Influenza A/WSN/33 Virus Infection Arrests Cell Cycle in G_1_ Phase

**FIGURE 5.**
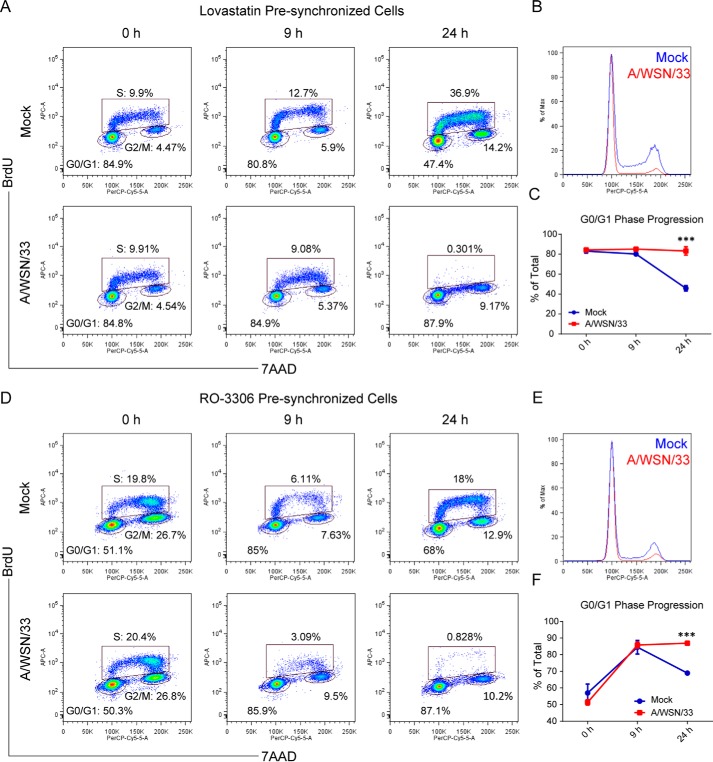
**IAV leads to cell cycle arrest in G_0_/G_1_.** *A*, IAV infection altered the cell cycle progression of A549 cells presynchronized in G_1_ phase. A549 cells were treated with lovastatin 22 h before infection with A/WSN/33 virus to synchronize them in G_0_/G_1_ phase. Cells were released from drug treatment at the start of infection (MOI = 2), which lasted for the indicated time periods, and incubated with 10 μm BrdU for cell cycle analysis 1 h before harvesting. *B*, cell cycle pattern of mock- and virus-infected cell populations at 24 h after infection that were presynchronized in G_0_/G_1_ phase with lovastatin. Results are representative of three independent experiments. *C*, percentage of cells in G_0_/G_1_ phase after lovastatin treatment in mock- or virus-infected cells. Line graph illustrates mean ± S.D. (*error bars*) of three independent experiments. ***, *p* < 0.001 *versus* mock at each time point by two-way ANOVA. *D*, IAV infection arrested cells in G_0_/G_1_ phase in an A549 population presynchronized in G_2_/M phase. CDK1 inhibitor RO-3306 was used to accumulate A549 cells in G_2_/M for 18 h. Immediately after drug removal, cells were infected with A/WSN/33 virus (MOI = 2) and treated as above for cell cycle analysis following BrdU incorporation. *E*, cell cycle pattern of mock- and virus-infected cell populations that were presynchronized in G_2_/M phase with RO-3306. Results are representative of three independent experiments. *F*, percentage of cells in G_0_/G_1_ phase after RO-3306 treatment in mock- or virus-infected cells. The line graph illustrates mean ± S.D. of three independent experiments. ***, *p* < 0.001 *versus* mock at each time point by two-way ANOVA. Flow cytometry data were obtained with a BD LSRII flow cytometer. Scattergrams are representative of three independent experiments. In *A* and *D*, scattergrams show DNA content staining *versus* BrdU incorporation of each individual cell for cell cycle status determination. *Boxed* and *circled areas* define cell populations in each phase of the cell cycle indicated on the graph.

The principal role of the D-type cyclins is to act as growth factor sensors, converting environmental signals into fuel for the cell cycle engine. The interaction between cyclin D3 and M2-CT during the viral life cycle implies a potential regulation of host cell cycle by IAV. This is supported by our observation that cyclin D3 is actively removed from the nucleus and degraded by the proteasome in virus-infected cells. To address whether the cell cycle is compromised, a quantitative profiling of cell cycle kinetics was performed on virus-infected A549 cells. Using an asynchronously growing cell population, we found that virus-infected cells accumulated at G_1_ phase when compared with the uninfected group (data not shown). To reduce variability due to a mixed population of cells in differential phases of the cell cycle, we first synchronized them before infection in G_1_ using lovastatin, a fungal metabolite shown to affect cell cycle distribution in a reversible manner (55). Cells were released from the lovastatin block and stimulated with mevalonate at the time of infection to trigger progression into later cell cycle stages. BrdU incorporation and DNA content staining showed that virus infection brought about a cell cycle arrest at 9 h p.i. (Fig. 5*A*). The overlay of cell cycle patterns of virus-infected and uninfected cells indicates that infection decreased the peaks representing cells in S and G_2_/M phases (Fig. 5*B*). The percentages of cells in G_1_ phase exhibited a progressive, time-dependent drop from 83.0 ± 2.1 to 80.1 ± 0.9% and subsequently 45.7 ± 2.8% (at 0, 9, and 24 h p.i., respectively; means ± S.D. of three experiments) in the mock-infected samples. This reduction reflects progression of cells through G_1_ phase, followed by entry into the later stages of the cell cycle. In contrast, cells infected by A/WSN/33 virus remained in G_1_ with the percentage of cells in G_1_ phase hovering above 80% at all time points (Fig. 5*C*). Our results demonstrate that IAV infection impairs progression of cell cycle through the G_1_/S intersection.

To further prove that IAV infection induced cell cycle arrest in G_1_ phase, we used a separate approach. Cells were preaccumulated in G_2_/M phase by treatment with RO-3306, a CDK1 inhibitor (56). At 0 h p.i., both A/WSN/33-infected and control samples had comparable percentages of cells in all phases of the cell cycle (Fig. 5*D*). Similar to the result obtained with lovastatin, the cell cycle pattern of virus-infected cells showed lower peaks for both S and G_2_/M phases when compared with uninfected cells (Fig. 5*E*). Upon recovery of cells from drug restriction, both uninfected and infected cells resumed division and initiated a new round of cell cycle, displaying an increased percentage of cells representing G_0_/G_1_ phase at 9 h p.i.; only uninfected cells, however, progressed further into S phase. The percentage of cells in G_0_/G_1_ phase dropped from around 85 to 65% in the control sample, whereas it remained constant in IAV-infected cells (Fig. 5*F*). Collectively, these results indicate that IAV infection perturbs normal cell cycle progression in human cell lines and installs a block at G_0_/G_1_ phase.

**FIGURE 6.**
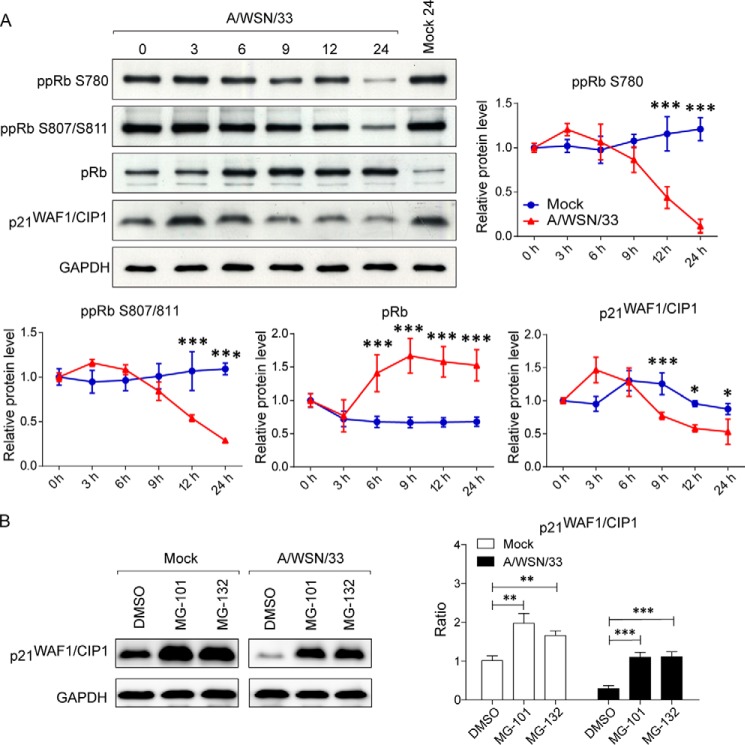
**IAV infection inhibits hyperphosphorylation of Rb and down-regulates CDK inhibitor p21^WAF1/CIP1^.** *A*, A549 cells were infected with A/WSN/33 virus at an MOI of 2. At the indicated time points, whole cell lysates were prepared from both infected and uninfected cells to determine the phosphorylation status of Rb and levels of CDK inhibitor p21^WAF1/CIP1^. Results are representative of three independent experiments. *Right*, densitometric analyses of Rb and p21^WAF1/CIP1^ levels were performed on immunoblots (normalized to GAPDH). Line graphs represent the mean ± S.D. (*error bars*) of three independent experiments. *, *p* < 0.05; **, *p* < 0.001 *versus* mock at each time point by two-way ANOVA. *B*, p21^WAF1/CIP1^ is targeted for proteolysis after viral infection. *Left*, A549 cells, either mock- or virus-infected, were treated with proteasome inhibitor MG-101 or MG-132 at the initiation of viral infection. Whole cell lysates were extracted at 9 h after infection for detection of p21^WAF1/CIP1^ protein levels. *Right*, densitometric analysis of immunoblots for p21^WAF1/CIP1^ levels in cells treated with different inhibitors (normalized to GAPDH). Results are representative of three independent experiments. The bar graph shows the mean ± S.D. from three independent experiments. **, *p* < 0.005; ***, *p* < 0.0005 by unpaired Student's *t* test.

D-type cyclins activate CDK4/6, which initiate phosphorylation of Rb at multiple sites. Hyperphosphorylated Rb (ppRb) releases transcription factor E2F and leads to the activation and transcription of cell cycle progression-responsive genes (38, 57, 58). To test whether IAV-induced cell cycle arrest was imposed by Rb, the phosphorylation status of Rb was monitored. Phosphorylation on serine 780 (ppRb Ser-780) is specifically catalyzed by the cyclin D-CDK4/6 complex but not cyclin E-CDK2 (59). Not surprisingly, IAV infection progressively resulted in diminished levels of ppRb Ser-780 as a function of time (Fig. 6*A*). Furthermore, serine 807/811 phosphorylation on Rb (ppRb Ser-807/811), which affects the S phase of the cell cycle displayed a similar trend (60) (Fig. 6*A*), an observation consistent with the virus-induced block at G_0_/G_1_ phase. In line with the down-regulation of ppRb proteins, accumulation of hypophosphorylated Rb (pRb) was observed (Fig. 6*A*).

Inhibition of cell cycle can also occur through induction of cyclin-dependent kinase inhibitors (CKIs). P21^WAF1/CIP1^ is a “universal” CKI that inhibits both cyclin and CDK subunits to install a p53-dependent G_1_ phase arrest (61). Interestingly, upon infection, p21^WAF1/CIP1^ was down-regulated through proteasomal degradation, indicating that IAV-induced G_0_/G_1_ arrest was not mediated through the p53/p21^WAF1/CIP1^ pathway (Fig. 6, *A* and *B*). Expression levels of p16 from the INK4 family of CKI, which specifically inhibits the catalytic subunits of CDK4 and CDK6, were also measured. No increase in p16^INK4A^ was observed even after prolonged infection time (data not shown).

From the results described above, we conclude that IAV infection down-regulates cyclin D3 levels, which in turn inhibits CDK4/6 activation and ultimately results in reduced Rb phosphorylation. Consequently, pRb sequesters the transcription factor E2F, which prevents cell cycle entry into S phase (58).

##### Cyclin D3 Negatively Modulates Influenza Virus Infection

**FIGURE 7.**
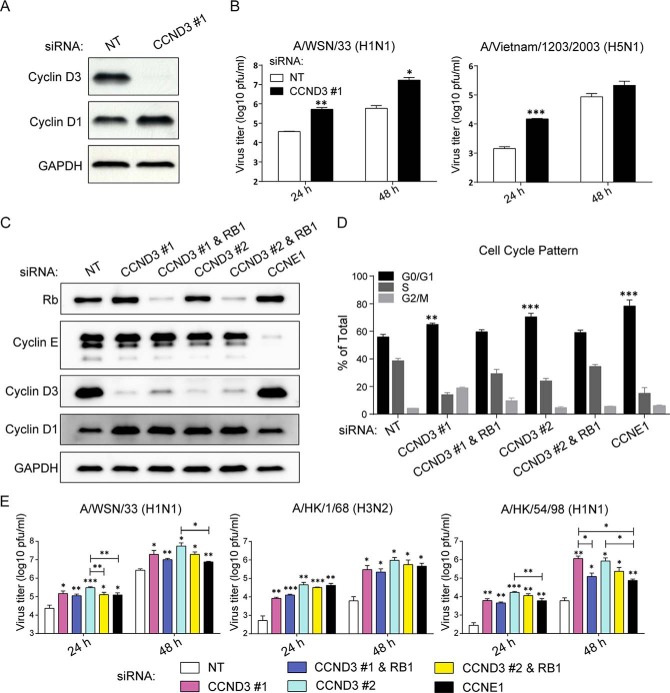
**Cyclin D3 negatively modulates influenza virus infection.** *A*, analysis of siRNA-mediated gene knockdown efficiency. Whole cell lysates were prepared from A549 cells 72 h after the designated siRNA treatment. Western blotting was used to determine the expression levels of cyclin D3 and cyclin D1. Results are representative of three independent experiments. *B*, *Ccnd3* knockdown in A549 cells facilitates both low pathogenic and highly pathogenic IAV infection. NT or cyclin D3 (CCND3 #1 and CCND3 #2) siRNA-treated cells were infected with viruses at an MOI of 0.01 for multicycle infection. Progeny virus titers in cell culture supernatants were determined through a plaque assay. The bar graphs depict the mean ± S.D. (*error bars*) of three independent experiments. *, *p* < 0.05; **, *p* < 0.01; ***, *p* < 0.0001 *versus* control (NT) siRNA at each time point by unpaired Student's *t* test. *C*, analysis of siRNA-mediated gene knockdown efficiency. Lysates were prepared from A549 cells treated with the indicated siRNAs 72 h post-transfection. The expression levels of Rb, cyclin E, cyclin D3, cyclin D1, and GAPDH proteins were determined by Western blotting. Results are representative of three independent experiments. *D*, cell cycle pattern of siRNA-treated cells. A549 cells were fixed in 70% ethanol 72 h after siRNA treatment. The cell cycle pattern of each group was quantitated through BrdU incorporation and 7-aminoactinomycin D DNA content staining. Data were obtained with a BD LSRII flow cytometer and presented as the percentage of total cells in each phase. The bar graph shows the mean ± S.D. of three independent experiments. **, *p* < 0.001; ***, *p* < 0.0001 *versus* NT siRNA by unpaired Student's *t* test. *E*, cyclin D3 depletion in A549 cells increases progeny virus titer in supernatant. A549 cells treated with the indicated siRNAs were infected with influenza A/WSN/33 (H1N1) virus, A/HK/1/68 (H3N2), or A/HK/54/98 (H1N1) virus at an MOI of 0.01. Cell culture supernatants were collected at 24 and 48 h after infection, and virus titers were determined by a plaque assay on MDCK cells. The bar graphs show the mean ± S.D. of three independent experiments. *, *p* < 0.05; **, *p* < 0.01; ***, *p* < 0.0005 *versus* NT siRNA by unpaired Student's *t* test.

To establish a functional relevance for cyclin D3 in IAV pathogenesis, we studied the effect of *Ccnd3* knockdown on virus replication. To avoid off-target effects imposed by the use of siRNA pools, we used an individual siRNA duplex from the published literature (CCND3 #1) for generating a knockdown (62). siRNA CCND3 #1 effectively reduced endogenous cyclin D3 expression levels by >90% at 72 h post-transfection, compared with a nonspecific control siRNA (nontargeting (NT)) (Fig. 7*A*). Control (NT siRNA-treated) and cyclin D3-deficient cells were infected with A/WSN/33 at an MOI of 0.01 to allow multiple rounds of infection. Viral titers obtained from cyclin D3-deficient cells were at least 10-fold greater than those from control cells (Fig. 7*B*, *left*). Because cyclin D3-M2 interaction was identified using avian M2-CT, we measured the effect of *Ccnd3* knockdown on the replication of highly pathogenic H5N1 avian influenza virus and observed a more >10-fold higher progeny virus production in cyclin D3-deficient cells when compared with control group at 24 h p.i. (Fig. 7*B*, *right*). The difference in production of H5N1 virus was less significant at 48 h p.i. (∼2.5-fold), most likely due to the cytopathic effect of H5N1 virus in a limited cell culture system.

To differentiate whether the increased progeny virus titer was intrinsic to the absence of cyclin D3 protein or a consequence of cell cycle arrest induced by cyclin D3 silencing, we next used combinations of siRNAs targeting various steps in the cell cycle to modulate its progression. Concomitant knockdown of *Ccnd3* and *Rb1* rescued cells from the G_0_/G_1_ phase block imposed by cyclin D3 deficiency, allowing us to measure the effect of cyclin D3 alone on virus replication. In addition, we included a *Ccne1* knockdown, which induced cell cycle arrest at the G_1_/S interface without affecting cyclin D3 levels. To gain further confidence in the specificity of the cyclin D3 siRNA, we included a second siRNA duplex specific for cyclin D3 (CCND3 #2). Both individual siRNAs and siRNA combinations resulted in significant reduction of the desired gene expressions (Fig. 7*C*). In the control siRNA-treated cell population, around 55% of cells were found in G_0_/G_1_ phase of the cell cycle. As expected, knockdown of *Ccnd3* alone arrested cell cycle progression in A549 cells with ∼65% (CCND3 #1) or ∼70% (CCDN3 #2) of cells in G_0_/G_1_ phase when compared with cells treated with NT siRNA. A concomitant *Ccnd3*/*Rb1* knockdown rescued cell cycle progression into S phase. Knockdown of *Ccne1* expression in A549 cells induced a more robust cell cycle G_0_/G_1_ phase accumulation than *Ccnd3* knockdown with ∼80% cells in G_0_/G_1_ phase (Fig. 7*D*). For all siRNA-mediated knockdowns, cells were subjected to infection with A/WSN/33 virus at an MOI of 0.01 at 72 h post-transfection for progeny virus production. *Ccnd3* knockdown with the two individual siRNA duplexes increased progeny virus titers by approximately 1 log. Despite the rescue in cell cycle progression, a double knockdown of *Ccnd3*/*Rb1* did not normalize virus production when compared with NT siRNA-treated cells. A 5-fold difference in viral titers persisted between control cells and those that harbored a double knockdown in *Ccnd3*/*Rb1*. Virus titers from cells with *Ccne1* knockdown were comparable with those from *Ccnd3*/*Rb1* double knockdown (Fig. 7*E*, *left*).

To study whether the inhibitory role of cyclin D3 on viral replication is also applicable to the other IAV strains, two clinical isolates were tested. At both 24 and 48 h p.i., equivalent or higher virus titers for influenza A/HK/1/68 (H3N2) and A/HK/54/98 (H1N1) viruses confirmed our results with A/WSN/33 (Fig. 7*E*, *middle* and *right panels*).

**FIGURE 8.**
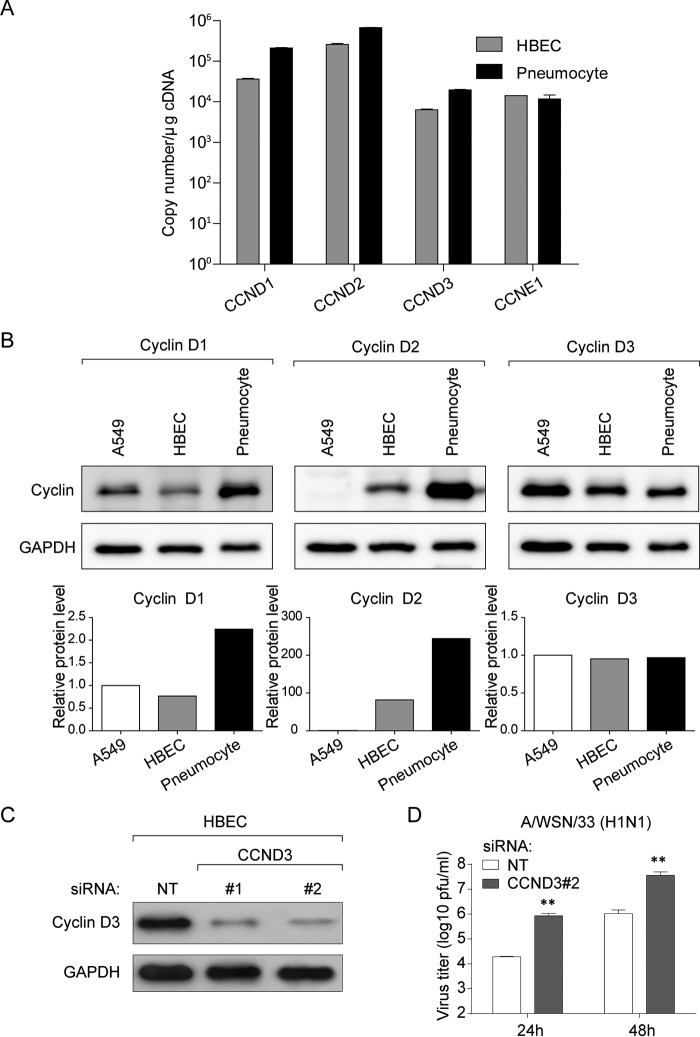
**Cyclin D3 is expressed along the human airway.** *A*, total RNA extracted from HBECs and pneumocytes was reverse transcribed into cDNA for Taqman probe-based quantitative PCR gene expression analysis. Shown is a graphical representation of the mean absolute copy number of cyclin D1, D2, D3, and E1 in HBECs and pneumocytes. *B*, whole cell lysates of A549 cells, HBECs, and pneumocytes were extracted to determine the protein levels of the designated cyclins through Western blotting (*top*). Densitometric analyses of immunoblots for cyclin D1, D2, and D3 in different cell types are shown in the *bottom panel* (normalized to GAPDH). *C*, analysis of siRNA-mediated gene knockdown efficiency in HBEC. Whole cell lysates were prepared from HBECs cells 72 h after the designated siRNA treatment. Western blotting was used to determine the expression levels of cyclin D3. *D*, *Ccnd3* knockdown in HBEC facilitates IAV progeny virus production. NT or cyclin D3 (CCND3 #2) siRNA-treated cells were infected with viruses at an MOI of 0.01 for multicycle infection. Progeny virus titers in cell culture supernatants were determined through a plaque assay. The bar graphs depict the mean ± S.D. (*error bars*) of three independent experiments. *, *p* < 0.05; **, *p* < 0.01; ***, *p* < 0.0001 *versus* control (NT) siRNA at each time point by unpaired Student's *t* test.

To examine the distribution of D-type cyclins in the context of natural infection, we determined their expression in the human airway, in particular human bronchial epithelial cells (HBECs) and pneumocytes. Although cyclin D3 mRNA was expressed at lower levels compared with the other two D-type cyclins in both cell types (Fig. 8*A*), comparable protein amounts of cyclin D3 were detected in A549, HBEC, and pneumocytes (Fig. 8*B*). Cyclin D2 expression could be detected in the two primary cells but not in A549 (Fig. 8*B*). Collectively, cyclin D3 is expressed in tissues along the natural infection route of IAV. To validate its functional relevance in these cells, we performed a knockdown of cyclin D3 in HBECs to study its effect on influenza virus production. As observed with A549 cells, cyclin D3 deficiency in HBEC resulted in ∼10-fold increase in viral titers, supporting our conclusions from experiments performed in actively dividing cells (Fig. 8, *C* and *D*).

##### Cyclin D3 Inhibits M2 and M1 Interaction but Not Viral Protein Synthesis

**FIGURE 9.**
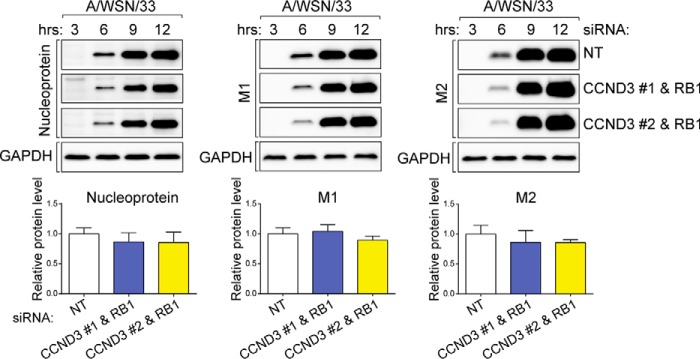
**Cyclin D3 deficiency does not affect viral protein synthesis.** *Top*, A549 cells with knockdown of the indicated genes were infected with A/WSN/33 virus at an MOI of 2 to determine their effect on viral protein synthesis. Whole cell lysates were collected at 3, 6, 9, and 12 h after infection to monitor protein levels. Lysates were resolved by SDS-PAGE, and levels of viral nucleoprotein, M1, and M2 were determined through Western blotting. Results are representative of three independent experiments. *Bottom*, densitometric analyses of immunoblots for each protein at 12 h p.i. (normalized to GAPDH). Results are shown as the mean ± S.D. (*error bars*) of three independent experiments.

To understand the mode of cyclin D3-imposed restriction of IAV production, we first measured whether viral protein synthesis was affected by cyclin D3. To separate the effect of cyclin D3 deficiency from cell cycle arrest, we used the *Ccnd3*/*Rb1* double knockdown. Multiple time points were selected to measure viral protein synthesis within a single cycle of infection. Knockdown cells did not display enhanced viral protein synthesis compared with control cells (Fig. 9), suggesting that the effect of cyclin D3 on IAV infection probably occurred at a later step after viral protein translation.

**FIGURE 10.**
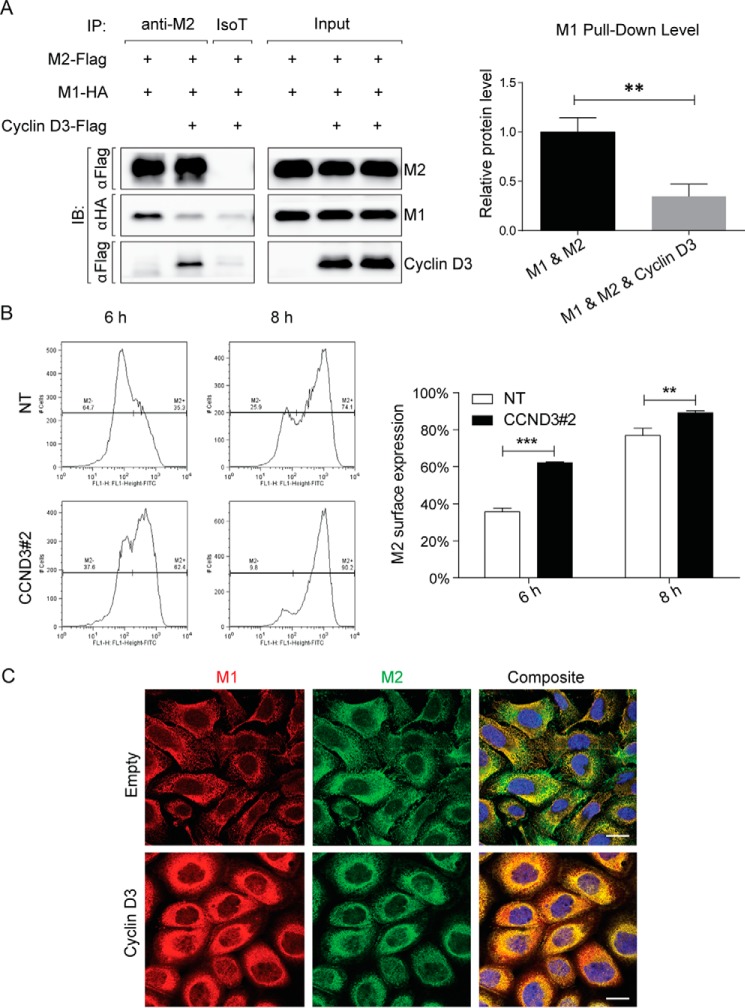
**Cyclin D3 competes with M1 for M2 binding.** *A*, cyclin D3 impairs interaction between viral M2 and M1 proteins. *Left*, HEK 293T cells were co-transfected with M1-HA and M2-FLAG (*lane 1*) or with M1-HA and M2-FLAG along with cyclin D3-FLAG (*lanes 2* and *3*), as indicated *above* each *blot*. At 48 h post-transfection, cell lysates were collected and subjected to immunoprecipitation using anti-M2 (*lanes 1* and *2*) or isotype control (*lane 3*) antibodies. Precipitates were analyzed through Western blotting to detect M1, M2, and cyclin D3 proteins. *Right*, densitometric analyses of immunoblots to quantitate the amount of viral M1 protein co-immunoprecipitating with M2 with or without the expression of cyclin D3 (normalized to immunoprecipitated M2 amount). Results are shown as mean ± S.D. (*error bars*) of three independent experiments. **, *p* < 0.005 by unpaired Student's *t* test. *B*, cyclin D3 hinders M2 cell surface expression. *Left*, control and cyclin D3-deficient cells were infected with A/WSN/33, and plasma membrane expression of viral M2 protein was detected by flow cytometry at 6 and 8 h after infection. Histograms are representative of three independent experiments. Gating indicates cell populations that are positive or negative for plasma membrane expression of M2 protein. *Right*, statistical analysis of the percentage of cells that were positive for surface expression of M2. Results are shown as mean ± S.D. of three independent experiments. **, *p* < 0.005; ***, *p* < 0.0001 by unpaired Student's *t* test. *C*, overexpression of cyclin D3 sequestered viral M1 and M2 protein to the secretory network. A549 cells were overexpressed with cyclin D3 protein 24 h before infection with A/WSN/33 virus at an MOI of 2. Cells were fixed at 10 h p.i. and immunostained with specific antibodies to reveal the subcellular localization of M1 (*red*) and M2 (*green*). *Bar*, 20 μm.

The biogenesis of viral particles involves proper packaging and assembly of viral proteins and RNA to form intact virions at discrete budding sites. M2 is a multifunctional protein; its cytosolic tail interacts with M1, promoting the recruitment of viral internal proteins and vRNA to the plasma membrane for efficient virus assembly (27). We postulated that binding of cyclin D3 to M2-CT might affect M1-M2 interaction, resulting in attenuated virus release due to either (i) production of defective virus particles due to improper packaging or (ii) incomplete assembly of virions and hence generation of fewer particles. To test this hypothesis, HEK 293T cells were co-transfected with cyclin D3, M1, and M2. Cells expressing M1 and M2 without exogenous cyclin D3 were used as control. For both conditions, M1-M2 binding was detected through co-immunoprecipitation followed by immunoblotting (Fig. 10*A*, *left*, *lane 1*). Upon overexpression of cyclin D3, the level of M1 that co-precipitated with M2 was significantly lower than the amount obtained in the absence of cyclin D3 (Fig. 10*A*, *left*, *lane 2*). Densitometric analysis revealed that a reduction of ∼70% in binding of M1 to M2 was observed when cyclin D3 was overexpressed (Fig. 10*A*, *right*). An isotype control antibody used in parallel did not bind to M1 (Fig. 10*A*, *left*, *lane 3*). Our results demonstrate that (i) cyclin D3 does not affect viral protein synthesis during IAV life cycle and (ii) interaction between M1 and M2 is significantly attenuated in the presence of cyclin D3. We propose that through competition with M1 for M2 binding, cyclin D3 restricts IAV production either through reduced or defective progeny virion formation.

To test this hypothesis, cyclin D3-deficient cells and control cells were infected with A/WSN/33 to measure plasma membrane expression of viral M2 protein within one cycle of infection. As illustrated in Fig. 6*C*, more M2 was detected at the plasma membrane in cyclin D3-deficient cells. This suggests that without the blockage imposed by cyclin D3, a greater amount of M2 could reach the cell surface and/or at a faster pace, thus facilitating the recruitment of viral internal components to the plasma membrane for assembly and budding. This trend could be observed at both time intervals, as summarized in Fig. 10*B*. In addition, we could validate these results using confocal imaging. Overexpression of cyclin D3 resulted in both M1 and M2 being predominantly confined to the early secretory network compared with control cells with endogenous cyclin D3 levels, where a significant fraction of M2 appeared at the plasma membrane (Fig. 10*C*). Interestingly, although the absence of cyclin E1 (a weak interactor of M2), facilitates IAV progeny virus production, this effect occurs through a different mechanism because M2 surface expression remained unaffected in cells depleted of cyclin E1 (data not shown).

## Discussion

The search for the interactome of M2 protein of IAV in host cells has been limited so far. Host proteins that M2 has been shown to exploit include Na^+^/K^+^-ATPase, heat shock protein 40 (Hsp40), and p58^IPK^ (inhibitor of protein kinase) (63–67). In addition, the amphipathic helix located within the M2-CT binds to cholesterol and alters the local membrane curvature at the budding zone while providing a scaffold for M1 polymerization and filament elongation (68). Withdrawal of cholesterol or disruption of the M2 amphipathic helix inhibits viral filament formation (25). We previously identified annexin A6 as a novel interactor with the M2-CT, which impairs budding and release of IAV at late stages of the virus life cycle (69). More recently, M2 was found to interact with the ATG8/LC3 family of ubiquitin-related proteins residing on the autophagosomal membranes (70, 71). A highly conserved LC3-interacting region in M2 promoted binding and relocalization of LC3 to the plasma membrane. This mechanism allowed subversion of autophagy and facilitated inter-organism viral transmission by enhancing the stability of progeny virions.

**FIGURE 11.**
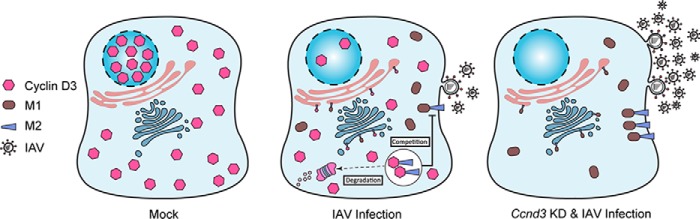
**Working model of cyclin D3 function in the context of influenza virus budding.** In normal cells, cyclin D3 displays nuclear localization, which is essential for cell cycle regulation. During influenza A virus infection, cyclin D3 interacts with viral M2 protein. The cyclin D3-M2 interaction impairs binding of M2 to M1, which is required for virus assembly and budding at the plasma membrane. In the absence of cyclin D3, more infectious progeny virus particles are released into the supernatant of virus-infected cells. Influenza virus, however, antagonizes cyclin D3 inhibition by (i) redistributing cyclin D3 between nucleus and cytoplasm, which contributes to cell cycle G_0_/G_1_ arrest, thus facilitating virus replication, and (ii) targeting cyclin D3 to the proteasomal degradation pathway in the cytoplasm to remove its interference with M1-M2 binding.

In the present study, we identified cyclin D3 as a novel interactor of IAV-M2 and systematically studied its role in the context of IAV infection. Being a key regulator of cell cycle progression, *Ccnd3* knockdown by default leads to cell cycle G_0_/G_1_ phase arrest, providing a favorable environment for IAV replication. Our results indicate that cyclin D3 functions beyond its role in cell cycle in the context of influenza infection. The absence of cyclin D3 in combination with accumulation of cell cycle in G_1_ phase appears to have an additive effect on IAV progeny virus production, indicating that cyclin D3 deficiency specifically facilitates influenza virus production independently of the cell cycle. Instead, co-immunoprecipitation with M2 suggests that cyclin D3 impairs nascent virus production at a late stage of the virus life cycle by interfering with M1-M2 binding (Fig. 11). Our results illustrate yet another mechanism that IAV has evolved to counteract host defenses; cyclin D3 is redistributed from the nucleus to the cytoplasm, where it is targeted for proteasomal degradation. Indeed, mislocalization and subsequent down-regulation of restriction factors is one of the recurring mechanisms viruses have adopted to overcome host defenses (72). The presence of M2 traps cyclin D3 in the cytoplasm through two possible mechanisms: (i) M2 interacts with newly synthesized cyclin D3 and prevents it from translocating into the nucleus, or (ii) cyclin D3 is retained by M2 in the cytoplasm after its nuclear export. A detailed mechanism of how cyclin D3 is targeted to the proteasome complex and which E3-ligase ubiquitylates it will be pursued in future investigations. Down-regulation of the cyclin D3 transcript was previously observed in H5N1-infected cells, but its implication had not been addressed (73). Our study has expanded the repertoire of IAV-M2 host interactors and proposes a novel function of cyclin D3 in the life cycle of IAV.

Manipulation of the cell cycle machinery has been adopted by many viruses as a common strategy. For instance, HIV-1 accessory protein Vpr is reported to induce arrest in G_2_ by activating protein phosphatase 2A, which acts on mitosis inhibitors Wee1 kinase and Cdc25 phosphatase (74). Similar to our findings, some other viral proteins have been found to bind members of the cyclin family. Persistent infection with borna disease virus results in reduced G_2_ to M progression (75). Likewise, the nucleoprotein of SARS-CoV inhibits the activity of CDK4 by binding to cyclin D, which blocks S phase progression (76). Viral proteins associate with particular cell cycle components, thereby deregulating the host cell cycle clock to alter the environment for more efficient replication. It remains to be determined whether M2-cyclin D3 interaction inhibits the kinase activity of CDK4/6 after IAV infection. A cell cycle arrest in G_0_/G_1_ by IAV infection was first reported by He *et al.* (49), although this phenomenon was not investigated in mechanistic terms. The non-structural protein 1 (NS1) of IAV was later discovered to interact with and inhibit the activity of the Ras homolog gene family member A (RhoA), providing some molecular insights into the mechanism of IAV-induced cell cycle perturbation (77). The present study quantitatively demonstrated that IAV infection imposes a specific G_0_/G_1_ arrest and established cyclin D3 mislocalization and degradation following its interaction with M2 as the underlying molecular correlate.

Co-evolution and adaptation of virus-host interactions is best described as a zero sum biological arms race. However, due to high mutation rates, viruses such as influenza are well equipped to rapidly evolve strategies to evade host restriction factors. Consequently, in the case of IAV infection, the effect of cyclin D3 is successfully antagonized by M2, which redirects cyclin D3 from the nucleus to the cytosol followed by proteasomal degradation, thereby granting an advantage to virus production.

Our study provides important insights into the mechanism through which cyclin D3 restricts IAV infection. We demonstrated that the increase in progeny virus production upon *Ccnd3* knockdown was not a result of enhanced viral protein synthesis in host cells. Instead, the inhibitory effect of cyclin D3 on IAV infection is due to its interaction with viral M2 protein, as measured by competitive co-immunoprecipitation assays. We hypothesize that cyclin D3-M2 interaction either masks the domain on M2 mediating binding to M1 or has a higher affinity than that between M1 and M2. Another possibility is that cyclin D3-M2 interaction sequesters the amount of available M2. Consequently, limited M1-M2 binding during the budding process of IAV infection results in fewer progeny virions being efficiently packaged and released from host cells. Several host factors have been identified that interfere with the late stage of the viral life cycle, among which tetherin (also known as BST-2) is the best characterized. In addition, interferon-stimulated gene (ISG) products are a group of newly emerging host restriction factors that can control viral, bacterial, and parasite infection, as exemplified by viperin and ISG15, to name a few (78–81). The current study includes cyclin D3 in the family of IAV budding inhibitors and provides insights into the dynamic interplay between cyclin D3 and M2 during IAV infection.

## Experimental Procedures

### 

#### 

##### Yeast Two-hybrid Screening

Influenza A virus M2-CT (A/Goose/Guangdong/1/96; GenBank^TM^ accession number 81975894) was used as bait to screen for interactors from a random-primed human placenta cDNA library, which contains 10 million independent fragments. The cloning of bait and Y2H screening were performed by Hybrigenics (Paris, France). Briefly, M2-CT was fused with the LexA binding domain by subcloning it into the pB27 vector. The L40ΔGAL4 yeast strain was then transformed with pM2-CT bait plasmid and mated with Y187 yeast strain transformed by the prey library (82). Clone selection was performed on leucine, tryptophan, and histidine drop-out medium, and 273 positive diploids were picked. Identified prey fragments were then subjected to PCR and sequencing. After going through filtration and being divided into contigs, sequences were compared with the most recently updated GenBank^TM^ database by using BLASTN to decode interactions. All interactions were assigned with computative confidence scores to assess interaction reliability (83).

##### Cells and Viruses

Human alveolar basal epithelial (A549), human embryonic kidney (HEK 293T), and Madin-Darby canine kidney (MDCK) cells were purchased from ATCC. Dulbecco's modified Eagle's medium (DMEM) (Invitrogen) supplemented with 10% fetal bovine serum (FBS), 100 units/ml penicillin, and 100 μg/ml streptomycin (Invitrogen) was used to culture and maintain all cell types used.

Influenza A virus strains A/WSN/33 (H1N1), A/HK/1/68 (H3N2), A/HK/54/98 (H1N1), and A/Vietnam/1203/2003 (H5N1) were propagated in MDCK cells supplemented with 0.3% bovine serum albumin (BSA) (Sigma-Aldrich) in the presence of 1 μg/ml tosylphenylalanyl chloromethyl ketone (TPCK)-treated trypsin (Thermo Scientific) and stored as virus stocks at −80 °C until further use. Viral titers were obtained by a plaque assay on MDCK cells. Briefly, serial 10-fold dilutions of virus stocks were adsorbed onto a confluent monolayer of MDCK cells for 1 h at 37 °C. Next, viral inoculums were removed, and cells were washed twice with phosphate-buffered saline (PBS), pH 7.4. Cells were then covered with 1% semisolid agar in DMEM complemented with 0.3% BSA and 1 μg/ml TPCK-treated trypsin. Three days after incubating the plates in an upside down manner, plaques were visualized by staining with crystal violet (84).

##### Plasmids and Antibodies

Plasmid pCMV6-FLAG-CCND3 was generated by subcloning the CCND3 open reading frame (ORF) (*Homo sapiens*, GenBank^TM^ accession number NM_001760.4) from plasmid pCMV6-CCND3-GFP (Origene Technologies) into the pCMV6-Entry vector at the MluI and SgfI restriction sites. Plasmid pCAGGS-M2-FLAG, pCAGGS-M1-HA, and rabbit anti-M1 polyclonal antibody (pAb) were kindly donated by Prof. Adolfo García-Sastre (Icahn School of Medicine at Mount Sinai).

The following primary antibodies were used in this study. Mouse anti-cyclin D3 monoclonal antibody (mAb) (clone DCS22) was purchased from Cell Signaling Technology; mouse anti-cyclin E mAb (clone HE12), mouse anti-influenza A M2 mAb (clone 14C2), mouse anti-influenza A M1 mAb (clone GA2B), and rabbit anti-p21 pAb were purchased from Santa Cruz Biotechnology, Inc.; mouse anti-influenza A M2 mAb (clone 14C2) was purchased from Thermo Scientific; mouse anti-FLAG mAb (clone M2) and mouse anti-c-Myc mAb (clone 9E10) were purchased from Sigma-Aldrich; mouse anti-human pRb mAb (clone G99-549), mouse anti-Rb (ppRb Ser-780) mAb (clone J146-35), and mouse anti-Rb (ppRb Ser-807/Ser-811) mRb (clone J112-906) were purchased from BD Biosciences; rabbit anti-cyclin D3 mAb (clone EP463E), mouse anti-influenza A nucleoprotein mAb (clone AA5H), mouse anti-lamin B1 mAb (clone ZL-5), and mouse anti-GAPDH mAb (clone 6C5) were purchased from Abcam; and Texas Red-conjugated goat anti-rabbit IgG (H+L) (pAb) and FITC-conjugated goat anti-mouse IgG (H+L) (pAb) were purchased from Life Technologies, Inc.

##### GST Pull-down

A DNA fragment encoding the M2 gene of influenza virus A/WSN/33 strain was cloned into the pGEX4T-1 vector and expressed in *Escherichia coli* strain BL21. The resulting GST fusion protein was purified with glutathione-Sepharose beads (GE Healthcare) and added to A549 cell lysates. The empty vector expressing GST alone was used as negative control. After overnight incubation, beads were collected by centrifugation and washed five times to remove nonspecific binding. Bound proteins were resolved by SDS-PAGE and immunoblotting with the indicated antibodies.

##### Co-immunoprecipitation

HEK 293T cells were transfected with pCMV6-FLAG-c-Myc-CCND3 plasmid in a 10-cm dish. At 48 h post-transfection, cells were infected with influenza A/WSN/33 strain at an MOI of 2 for 6 h before being lysed in buffer containing 50 mm Tris (pH 7.4), 150 mm sodium chloride, 1% Triton X-100, 0.5% sodium deoxycholate, 1 mm EDTA, and protease inhibitors (Roche Applied Science). Cell extracts were cleared by centrifugation, and supernatant was incubated with anti-FLAG or isotype control antibody overnight at 4 °C. Protein G-Sepharose beads (GE Healthcare) were then used to recover the immune complexes from whole cell lysates. Immunoprecipitated samples were washed five times with lysis buffer before being resolved by SDS-PAGE and detection by Western blotting. An interaction specificity study was performed in HEK 293T cells transfected with plasmid encoding any one of the c-Myc-tagged cyclins, D1, D2, D3, or E1, and targeted to similar treatment as described above for co-immunoprecipitation experiments.

For competitive binding assays, HEK 293T cells were co-transfected with pCAGGS-M1-HA and pCAGGS-M2-FLAG plasmids or with pCAGGS-M1-HA, pCAGGS-M2-FLAG, and pCMV6-FLAG-cMyc-CCND3 plasmids in 10 cm dishes. Bulk cell lysates were prepared as described above at 48 h post transfection. Cleared cell extracts were incubated with anti-M2 (Santa Cruz) or isotype control antibody overnight at 4 °C, followed by recovery of the immune complexes from whole cell lysates using protein G-Sepharose beads. Immunoprecipitated samples were washed six times with lysis buffer before being resolved by SDS-PAGE and detection by Western blotting.

##### Synchronization of Cells

Lovastatin was used to synchronize cells in G_1_ phase. Briefly, culture medium was removed 24 h after the initial plating of A549 cells and replaced with fresh complete medium containing 40 μm lovastatin (Sigma-Aldrich). Cells were incubated in lovastatin for 22 h to achieve cell cycle G_0_/G_1_ phase synchronization before other experimental procedures. At time 0, cells were allowed to reenter cell cycle progression by replacing the medium with fresh complete medium supplemented with 4 mm mevalonolactone (Sigma-Aldrich). The synchronization of cells at the G_2_/M transition by RO-3306 was as follows. At 24 h following plating A549 cells, medium was replaced with fresh complete medium containing 4 μm RO-3306 (Calbiochem) and incubated for 18 h. Cells were then washed three times with PBS and cultured in fresh complete medium to promote cell cycle progression.

##### Cell Cycle Analysis

Cell cycle profiles were analyzed by BrdU incorporation and DNA content staining. Briefly, presynchronized A549 cells were infected at an MOI of 2 with influenza A/WSN/33 strain or mock-infected. Immediately before collection, cells were incubated in 10 μm BrdU (Invitrogen) for 1 h and fixed overnight in 70% ethanol at −20 °C. BrdU-pulsed cells were rescued from ethanol and denatured by 2 m hydrochloric acid with 0.5% Triton X-100 for 30 min at room temperature, followed by neutralization with 100 mm borate buffer (pH 8.5). BrdU labeling was revealed with Alexa Fluor 647-conjugated BrdU mouse mAb (clone MoBU-1; Invitrogen). Total DNA content was measured by 7-aminoactinomycin D (BD PharMingen) staining. Cells were then immediately analyzed on a BD LSR II flow cytometer (BD Immunocytometry Systems). Approximately 30,000 cells were acquired for each sample, and data were analyzed using FlowJo software (Treestar).

##### Protein Analysis and Proteasome Inhibition Assay

A549 cells were infected with A/WSN/33 virus at an MOI of 2 or mock-infected. At various time points after infection, cells were washed twice with ice-cold PBS and lysed in buffer containing 50 mm Tris (pH 7.4), 150 mm sodium chloride, 1% Triton X-100, 0.5% sodium deoxycholate, 1 mm EDTA, and protease inhibitors. After end-to-end rotation at 4 °C for 1 h to facilitate lysis, cell lysates were cleared by centrifugation at 20,817 × *g* for 30 min at 4 °C. Whole cell lysates were further boiled at 95 °C for 10 min in 1× sample loading dye and 50 mm DTT before being subjected to SDS-PAGE.

To block ubiquitin-mediated protein degradation, A/WSN/33 virus-infected (MOI of 2) or mock-infected A549 cells were incubated in fresh medium containing either 40 μm MG-101 (Sigma-Aldrich) or 5 μm MG-132 (Sigma-Aldrich) at the start of infection. DMSO (Sigma-Aldrich)-containing medium was used as vehicle control in this experiment. Whole cell lysates were collected 9 h after infection as described above.

##### Subcellular Fractionation

For cell fractionation, A549 cells were infected at an MOI of 2 with influenza A/WSN/33 virus. At 8 h p.i., cells were washed twice with ice-cold PBS and lysed on a dish with ice-cold buffer containing 25 mm Tris (pH 7.4), 5 mm magnesium chloride, 150 mm sodium chloride, and 0.1% Triton X-100. Permeabilized cells were collected with a rubber policeman and transferred into Eppendorf tubes. After a very brief incubation, supernatant (cytoplasm-enriched) and pellet (nucleus-enriched) fractions were separated upon centrifugation at 20,817 × *g* for 30 s at 4 °C. The nuclear fraction was then solubilized in buffer containing 25 mm Tris (pH 7.4), 5 mm magnesium chloride, 150 mm sodium chloride, and 1% SDS. DNA shredder was used to process the nuclear fractions to shear the genomic DNA for easier SDS-polyacrylamide gel loading of the samples. Sample loading dye and DTT at a final concentration of 1× and 50 mm, respectively, were added into the lysate before boiling at 95 °C for 10 min.

##### Colocalization Assays

To capture subcellular colocalization between influenza M2 and cyclin D3, A549 cells were transfected with a wild-type cyclin D3 plasmid (pCMV6-CCND3-cherry). 24 h post-transfection, cells were either mock- or virus-infected with A/WSN/33 at an MOI of 2. Cells were maintained in medium supplemented with 5 μm MG132 for the duration of infection. 15 h p.i. cells were collected for the immunofluorescence assay.

To determine the effect of cyclin D3 on co-localization between influenza M1 and M2, A549 cells were transfected with either an empty vector or cyclin D3 (pCMV6-FLAG-CCND3). 24 h post-transfection, cells were infected with influenza A/WSN/33 at an MOI of 2. 10 h p.i., cells were collected for staining and confocal imaging of influenza M1 and M2.

##### Immunofluorescence Assay

A549 cells were plated onto glass coverslips 1 day before transfection or infection. Cells were washed with PBS and fixed in 4% paraformaldehyde for 15 min at the end of the experiments. Coverslips were incubated in 50 mm ammonium chloride for 10 min to quench the autofluorescence of paraformaldehyde before being permeabilized in PBS containing 0.1% Triton X-100 for 10 min. Cyclin D3 was stained with rabbit anti-cyclin D3 mAb (EP463E), followed by incubation with FITC-conjugated goat anti-rabbit IgG. The viral M2 protein was visualized by labeling with mouse anti-M2 mAb (14C2), followed by staining with Texas Red-conjugated goat anti-mouse IgG. The viral M1 protein was visualized by labeling with rabbit anti-M1 pAb, followed by FITC-conjugated goat anti-rabbit IgG. After washing with PBS, coverslips were incubated with DAPI for nucleus staining and mounted in Mowiol on glass slides. Images were acquired with a Zeiss LSM 700 confocal fluorescence microscope at the Faculty Imaging Core Facility of the University of Hong Kong.

##### RNA Interference and Virus Infection

All gene-specific siRNAs were purchased from Dharmacon (Dharmacon Research). Individual siRNAs against the 5′-AAGGAUCUUUGUGGCCAAGGA-3′ sequence (62) or 5′-UGCGGAAGAUGCUGGCUUA-3′ (D-003212-08) sequence of human cyclin D3 transcript and the nontargeting siRNA (D-001210) were used. siRNAs for human cyclin E1 transcript (M-003213) and human Rb1 transcript (M-003296) were provided as an siGENOME SMARTpool containing a mixture of four siRNAs targeting different sites of a single gene. A549 cells were transfected with siRNAs (final concentration of 100 mm for each gene) by using DharmaFECT 1 transfection reagent according to the manufacturer's protocol. Cells were subcultured 48 h post-transfection to avoid potential cell cycle arrest induced by overconfluence.

To study the effect of gene knockdown on influenza progeny virus production, siRNA-treated A549 cells or HBECs were infected with influenza viruses 24 h after seeding at an MOI of 0.01 in DMEM containing 0.3% BSA (Sigma-Aldrich), in the presence of 0.1 μg/ml TPCK-treated trypsin (Thermo Scientific). Supernatants were collected at 24 and 48 h p.i., and the progeny virus titers were obtained by a plaque assay as described above. Separate wells without infection were used to determine the knockdown efficiency by Western blotting.

The effect of siRNA treatment on influenza viral protein expression was investigated in cells prepared as described above. Cells were infected with A/WSN/33 virus at an MOI of 2 and supplied with fresh cell culture medium containing 10% FBS and 100 units/ml penicillin and 100 μg/ml streptomycin after removing the virus inoculums. Whole cell lysates were prepared from individual wells at 3, 6, 9, and 12 h p.i. for Western blotting analysis.

Cyclin D3-specific siRNA-treated and control A549 cells were infected at an MOI of 1 with A/WSN/33 virus to study the effect of cyclin D3 reduction on M2 plasma membrane expression. To synchronize viral entry, cells were incubated with virus at 4 °C for 1 h and replaced with prewarmed fresh cell culture medium. Virus-infected cells were collected and fixed at 6 and 8 h p.i. for surface M2 expression detection using anti-M2 mAb (Thermo Scientific) followed by FITC-conjugated secondary antibody without permeabilization. Cells were then immediately analyzed on a BD LSR II flow cytometer (BD Immunocytometry Systems). Approximately 30,000 cells were acquired for each sample, and data were analyzed using FlowJo software (Treestar).

##### Absolute Quantitative RT-PCR

Gene expression patterns were determined by two-step RT-PCRs. Briefly, total RNA was isolated from around 1 × 10^5^ HBECs and pneumocytes using an RNeasy Plus kit in combination with QIAshredder (Qiagen). cDNA was then generated from the total RNA using the SuperScript III first-strand synthesis system (Invitrogen) according to the manufacturer's recommendation. The cDNA product was used undiluted for HBECs or 20-fold diluted for pneumocytes. Serial dilutions of plasmids of cyclin D1, D2, D3, and E1 with known gene copy numbers were used to generate standard curves for absolute quantification of the desired gene expression. The real-time PCRs were performed in 25-μl reactions containing fluorigenic Taqman probe at a final concentration of 400 nm together with an 800 nm concentration of each primer and 12.5 μl of 2× Probe Master (Roche Applied Science). The assays were performed on the LightCycler 480 II (Roche Applied Science) with thermal cycling conditions of 10 min at 95 °C followed by 40 cycles of 95 °C for 10 s, 60 °C for 1 min, and 72 °C for 1 s. Each sample was quantified in triplicate for each experiment. Primers and probes were as follows: 5′-CTGTCCTACTACCGCCTCACA-3′ (forward), 5′-GCCTTGGGGTCCATGTTCT-3′ (reverse), and 5′-(FAM)CCAGAGTGATCAAGTGTGACCCAGACTGCC(TAMRA)-3′ (Taqman probe) for cyclin D1 (85); 5′-GGGAACTGGTGGTGCTGG-3′ (forward), 5′-TGGGTACATGGCAAACTTAAAGTC-3′ (reverse), and 5′-(FAM)AAGCTGTCTCTGATCCGCAAGCATGC(TAMRA)-3′ (Taqman probe) for cyclin D2 (85); 5′-TCCGGGGATGAGCTCACAGA-3′ (forward), 5′-GGCTCTCCCTGAGTGCAGCTT-3′ (reverse), and 5′-(FAM)TGGCAGGGATCACTGGCACTGAAGTGGACT(TAMRA)-3′ (Taqman probe) for cyclin D3 (85); 5′-GAAGATGATGACCGGGTTTAC-3′ (forward), 5′-GAGCCTCTGGATGGTGCAA-3′ (reverse), and 5′-(FAM)CAAACTCAACGTGCAAGCCTCGGAT(TAMRA)-3′ (Taqman probe) for cyclin E1 (86).

##### Statistical Analysis

Results are shown as mean ± S.D. of the indicated number of experiments. Statistical difference between groups was determined by unpaired Student's *t* test or two-way ANOVA, with a 0.05 significance level.

## Author Contributions

Y. F. designed, performed, and analyzed the experiments. C. K. P. M. performed the H5N1 experiment in Fig. 7. M. C. W. C. prepared the HBEC and pneumocyte samples. Y. Z. and F. K. provided technical assistance and contributed to the preparation of the figures. B. N. designed and supervised part of the experiments. R. B. and S. S. assisted Y. F. and analyzed the data and wrote the manuscript with input from all of the other authors.

## Footnotes

3 The abbreviations used are:

Y2H: yeast two-hybrid
TPCK: l-1-tosylamide-2-phenylethyl chloromethyl ketone
CT: cytoplasmic tail
CDK: cyclin-dependent kinase
IAV: influenza A virus
MOI: multiplicity of infection
p.i.: postinfection
Rb: retinoblastoma
ppRb: hyperphosphorylated Rb
pRb: hypophosphorylated Rb
CKI: cyclin-dependent kinase inhibitor
NT: nontargeting
HBEC: human bronchial epithelial cell
ISG: interferon-stimulated gene
contig: group of overlapping clones
MDCK: Madin-Darby canine kidney
pAb: polyclonal antibody
FAM: 6-carboxyfluorescein
TAMRA: tetramethylrhodamine
ANOVA: analysis of variance.
